# Responses to increasing exercise upon reaching the anaerobic threshold, and their control by the central nervous system

**DOI:** 10.1186/2052-1847-6-17

**Published:** 2014-04-24

**Authors:** Ana B Peinado, Jesús J Rojo, Francisco J Calderón, Nicola Maffulli

**Affiliations:** 1 Department of Health and Human Performance, Technical University of Madrid, Martín Fierro 7, 28040 Madrid, Spain; 2 Department of Musculoskeletal Surgery, University of Salerno School of Medicine and Surgery, Salerno, Italy; 3 Centre for Sports and Exercise Medicine, Queen Mary University of London, London, England

**Keywords:** Physiological response, Exercise, Central governor, Fatigue

## Abstract

The anaerobic threshold (AT) has been one of the most studied of all physiological variables. Many authors have proposed the use of several markers to determine the moment at with the AT is reached. The present work discusses the physiological responses made to exercise - the measurement of which indicates the point at which the AT is reached - and how these responses might be controlled by the central nervous system. The detection of the AT having been reached is a sign for the central nervous system (CNS) to respond via an increase in efferent activity via the peripheral nervous system (PNS). An increase in CNS and PNS activities are related to changes in ventilation, cardiovascular function, and gland and muscle function. The directing action of the central command (CC) allows for the coordination of the autonomous and motor systems, suggesting that the AT can be identified in the many ways: changes in lactate, ventilation, plasma catecholamines, heart rate (HR), salivary amylase and muscular electrical activity. This change in response could be indicative that the organism would face failure if the exercise load continued to increase. To avoid this, the CC manages the efferent signals that show the organism that it is running out of homeostatic potential.

## Introduction

The AT has been one of the most studied of all physiological variables [1-4]. In human studies, the interest of researchers has ranged from its better comprehension [5,6] to its use in medicine [7-9] and training [9-11].

The term ‘anaerobic threshold’ was coined by Wasserman and McIlroy [12] when using the respiratory exchange ratio (RER) to detect the beginning of anaerobic metabolism in patients with heart problems performing stress tests [12]. Later, Wasserman et al. [13] defined the AT as: 1) a non-linear increase in ventilation (Ve), 2) a non-linear increase in the elimination of CO_2_ (VCO_2_), 3) an increase in the end-tidal partial pressure of O_2_ during a series of breaths (PetO_2_) with no corresponding drop in the end-tidal partial pressure of CO_2_ (PetCO_2_), and 4) an increase in the RER with work load (all during an incremental exercise test) [13].

**Figure 1:**
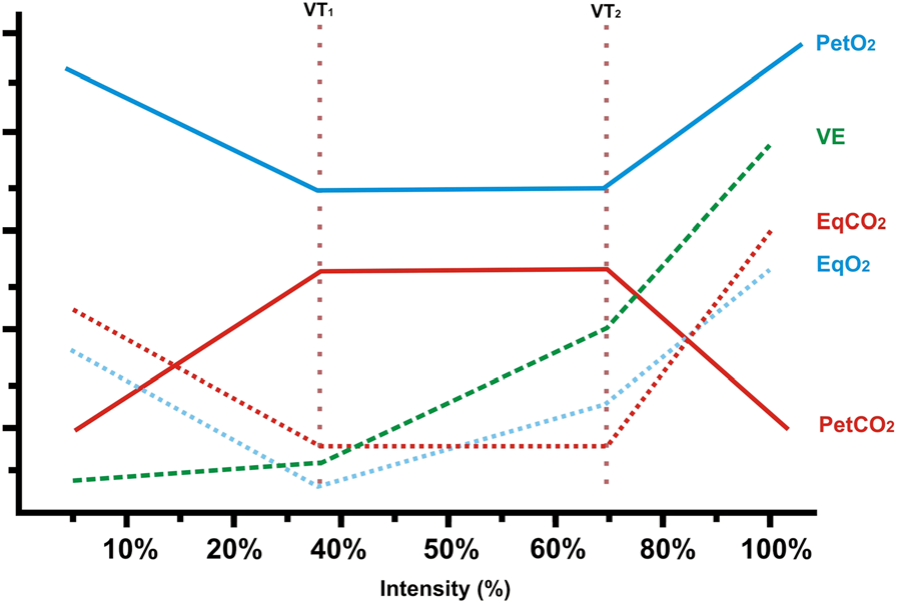
**Triphasic model described by Skinner and McLellan **[14]**.**

Skinner and McLellan [14] constructed the triphasic model (Figure 1), which uses gaseous exchange (breathing) variables and which distinguishes two thresholds: ventilatory threshold 1 (VT_1_) and ventilatory threshold 2 (VT_2_). According to these authors, VT_2_ is related to AT, since both are reached at the same time. Unfortunately, in the literature, VT_2_ is known by different names (Table 1), which has led to much confusion. Moreover, the definition of this phenomenon is clearly controversial as many different methodologies have been proposed to determine the AT.

**Table 1:** **Different terms used to refer to ventilatory threshold 2 as recorded by Orr [**[15]**]**

**Name**	**Authors**
Aerobic-anaerobic threshold	Mader and Heck (1976) [16]
Anaerobic threshold	Kinderman et al. (1979) [17]
Individual anaerobic threshold	Stegmann et al. (1981) [18]
Onset blood lactate accumulation (OBLA)	Sjodin and Jacobs (1981) [19]
Anaerobic threshold	Skinner and McLellan (1980) [14]

The VT_2_ is likely reached at the same time as the anaerobic lactate threshold (LT). The LT is the moment at which, during an incremental stress test, the plasma concentration of lactate increases above resting value. The intense and ongoing debate about this threshold is mainly based on terminology and/or the physiological background of LT concepts [20]. Thus, the AT, VT_2_ and LT are all supposed to be reached at the same time. However, debate still surrounds the exact relationship between VT_2_ and LT [21].

On the other hand, the mechanisms that regulate ventilation during exercise are not entirely understood [22]; the use of VT_2_ for the determination of AT, may, therefore, not be entirely reliable. Many authors therefore propose the use of other markers to determine the moment at with the AT is reached. For example, Davis et al. [23] proposed the determination of this point via the increase in plasma catecholamines (AT_catecholamines_) during an incremental exercise test. Yet others have proposed determining the electromyographic threshold (AT_EMG_) [24]. Others still have proposed measuring the concentration of salivary amylase (AT_saliva_) [25]; it is proposed that when the AT is reached, a change in the concentration of this enzyme becomes noticeable. Conconi et al. [26] proposed that a change in slope of the relationship between heart rate (HR) and exercise intensity (AT_Conconi_) be used as a marker of AT, although this remains controversial and several authors concluded that most haemodynamic variables, including HR, are unsuitable for indirect assessment of the AT [27].

**Figure 2:**
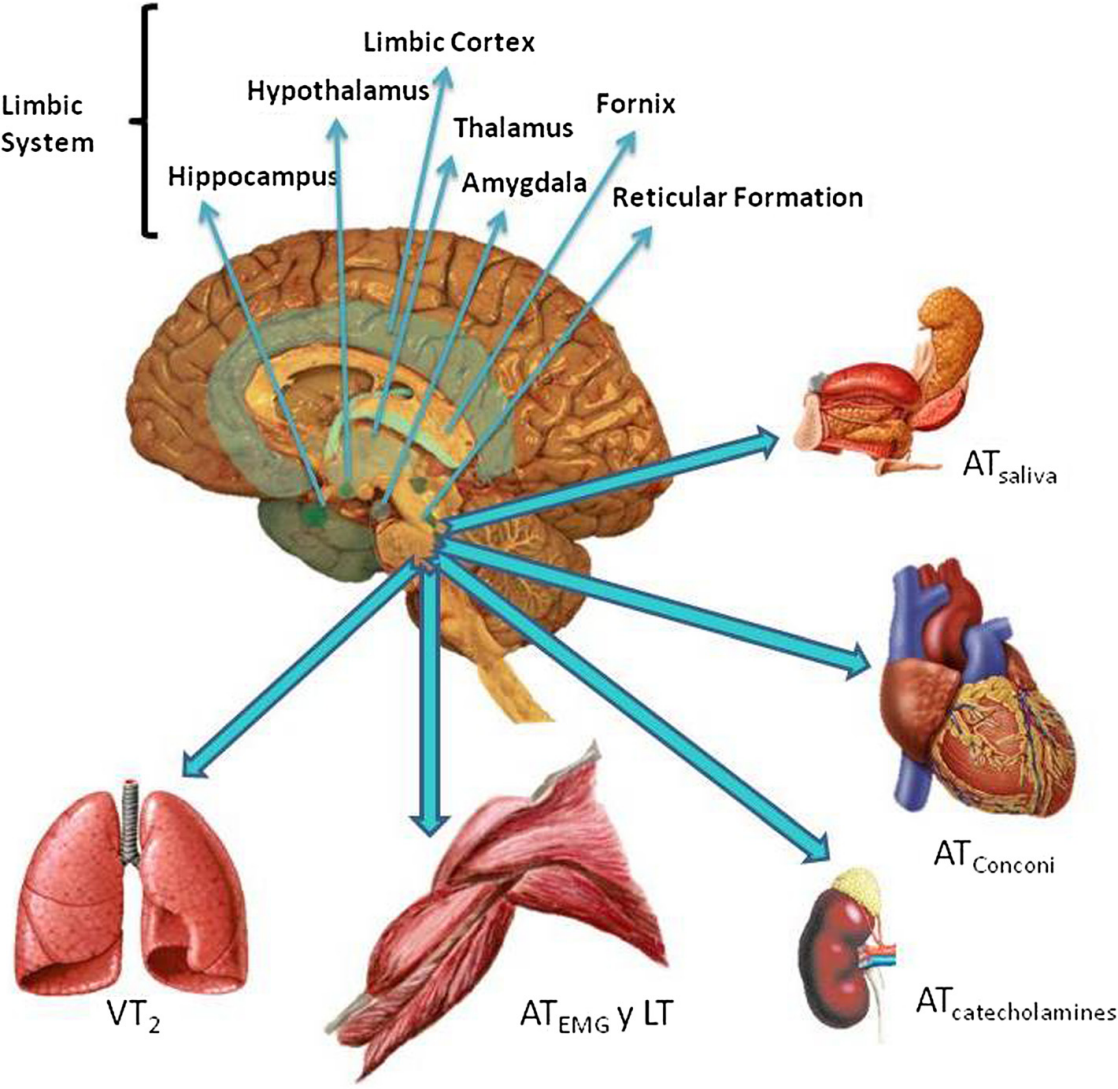
**Proposed relationship between the nervous system and the different means of determining that the AT has been reached.** AT_saliva_: saliva threshold; AT_catecholamines_: catecholamines threshold; AT_EMG_: electromyographic threshold; AT_Conconi_: heart rate threshold proposed by Conconi; VT_2_: ventilatory threshold 2; LT: lactate threshold.

The present work discusses the physiological responses made to exercise - the measurement of which indicates the point at which the AT is reached - and how these responses might be controlled by the central nervous system (CNS) (Figure 2). Many questions remain as to how the CNS coordinates responses to the AT having been reached. What type of information is processed by the CNS in order for it to respond? How is this information analysed?

### Detecting the different signals that the anaerobic threshold has been reached, and mounting a response

The detection of the AT having been reached is a sign for the CNS to respond via an increase in efferent activity via the peripheral nervous system (PNS). This response is regulated by the reticular formation, with the involvement of the brainstem nuclei and the limbic system. The limbic system maintains bi-directional communication (afferent and efferent) with the CNS, and participates in autonomic regulation via the voluntary and autonomous nervous systems. This probably alerts the entire organism that the AT has been reached and that a response is required. Figure 2 shows how an increase in CNS and PNS activities are related to changes in ventilation, cardiovascular function, and gland and muscle function.

#### Changes in different systems upon reaching the AT

**Figure 3:**
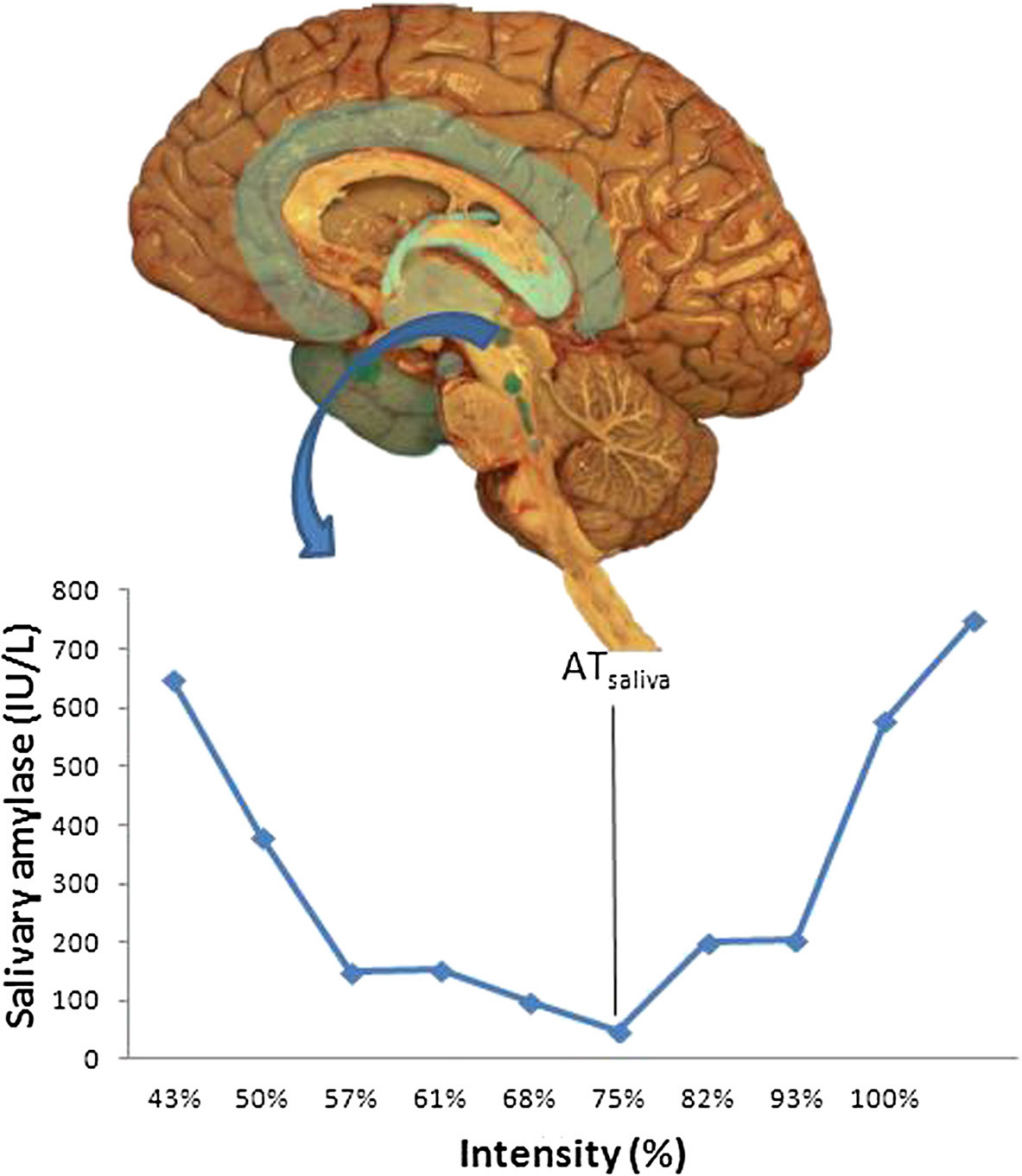
**Salivary amylase concentration during an incremental exercise test **[25]**.** AT_saliva_: saliva threshold.

During exercise the composition of the saliva may change via an increase in sympathetic activity. Indeed the AT can be pinpointed via the AT_saliva_. In humans, there are two types of salivary gland: the major glands (including the parotid, submaxillary and sublingual glands), and the minor glands (lingual glands). The major glands are controlled by the salivatory nuclei (superior and inferior) which are close to the dorsal nucleus of the vagus nerve, above the pontobulbar union [28]. These glands are innervated by sympathetic and parasympathetic neurons. The parotid glands receive sympathetic fibres from the external carotid plexus, along with parasympathetic fibres via the tympanic branch of the glossopharyngeal nerve, following synapses in the otic ganglion [28]. The afferent fibres of the salivary nuclei have not been well identified. Figure 3 shows the changes that occur in salivary amylase when the AT is reached [25].

**Figure 4:**
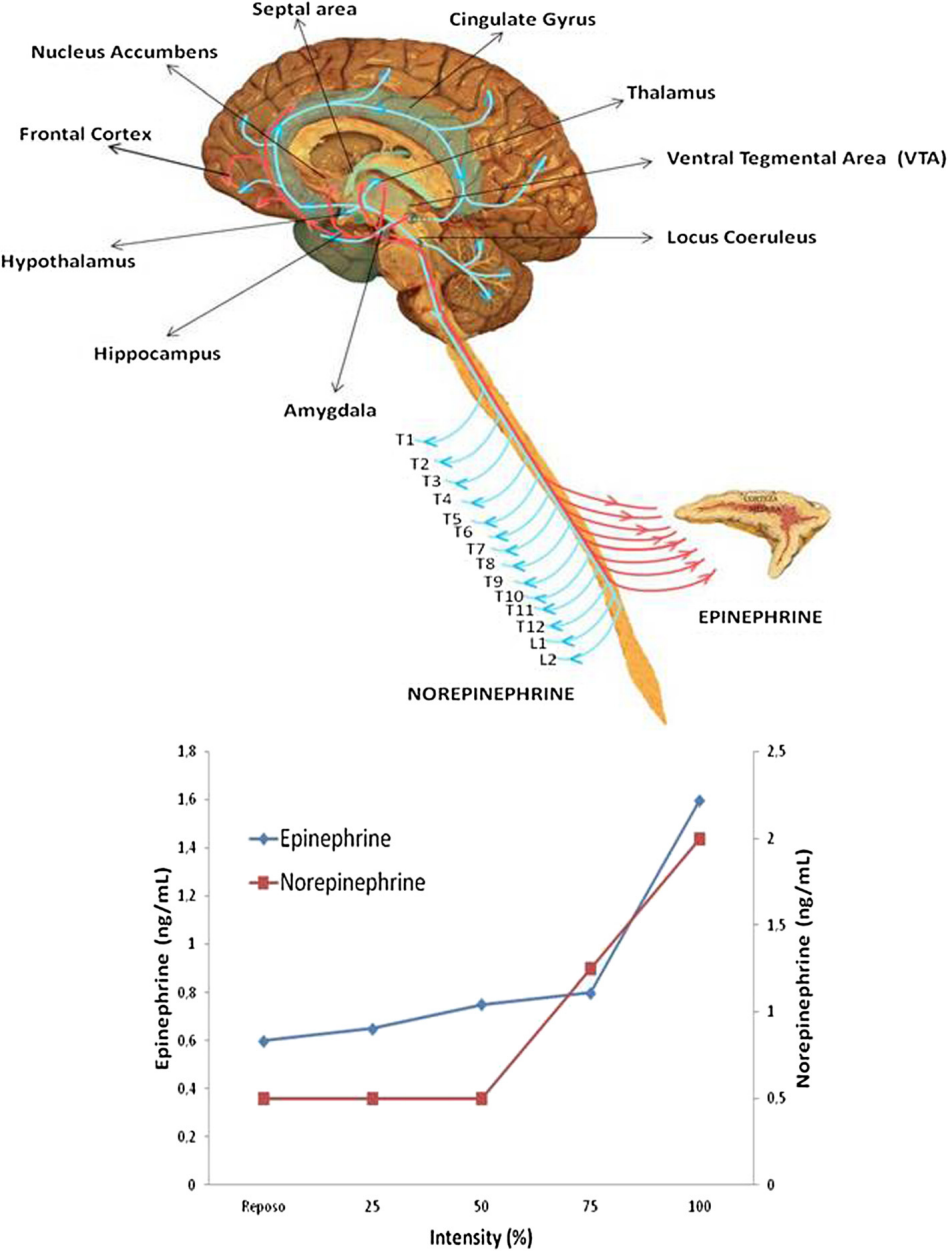
**Catecholamine release during an incremental exercise test.** Data unpublished.

During exercise the plasma concentration of adrenaline and noradrenaline may change. These hormones are secreted by the non-myelinated postsynaptic terminals of the sympathetic nervous system, and the medulla of the adrenal gland. The latter is innervated by the myelinated preganglionic fibres of the suprarenal plexus (formed by branches of the coeliac ganglion and the major splanchnic nerve) [28]. For their size, the autonomic innervation of the chromaffin cells is greater than that of any other type of cell. The nervous activity of thoracic segments 5–12, the intermediate neurones of which connect to the chain of corresponding sympathetic ganglia, terminate in the coeliac ganglion via the major and minor splanchnic nerves, and are responsible for the activation of the adrenal gland. Figure 4 shows how the increase in sympathetic nervous activity leads to an increase in catecholamine release. The reticular formation in the brainstem and spinal cord (which are controlled by the prosencephalic level of the limbic system) regulates the response.

**Figure 5:**
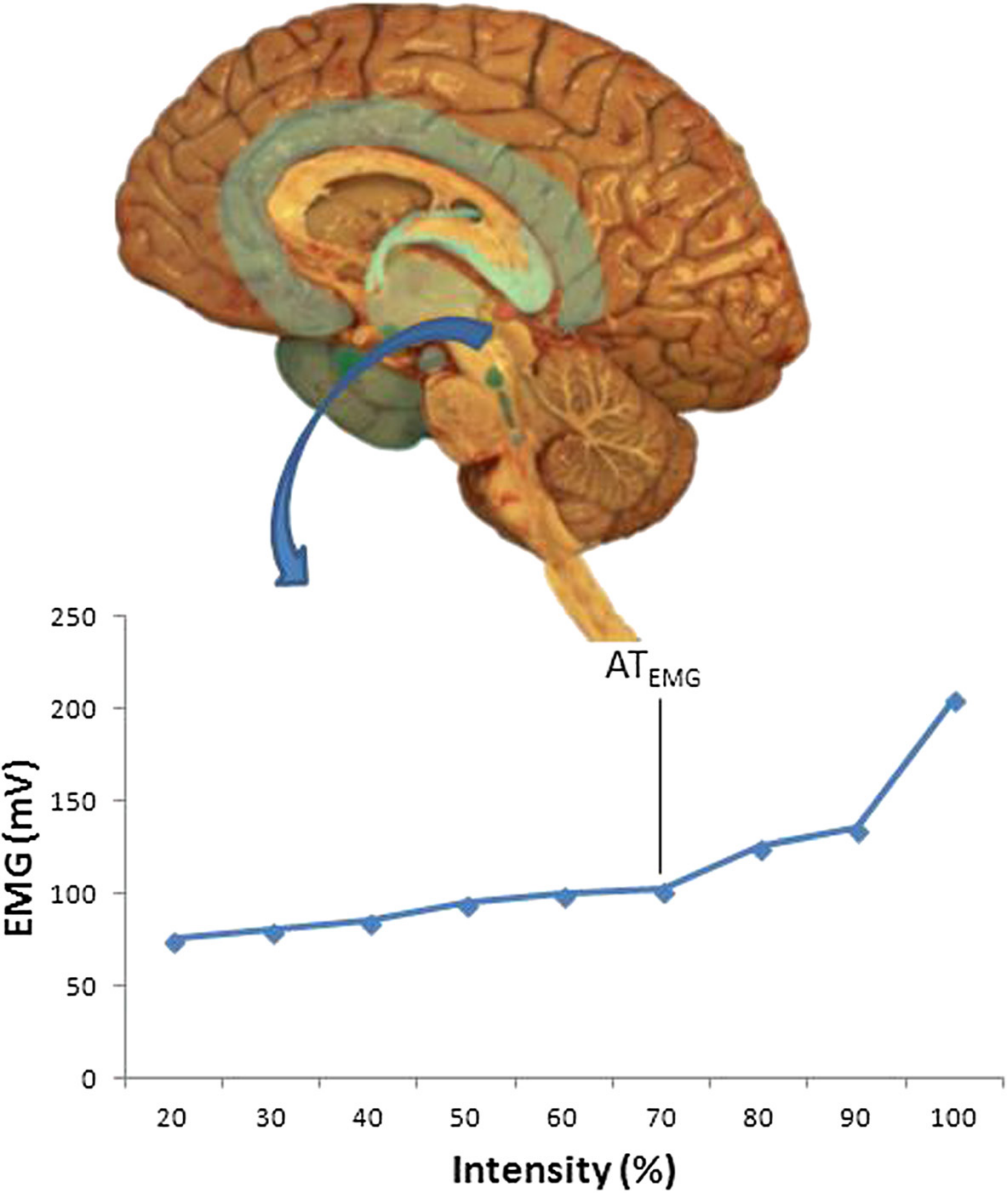
**Muscular electrical activity changes during an incremental exercise test.** Data unpublished. EMG: electromyography.

Finally, muscular electrical activity changes during exercise (Figure 5), along with the lactate concentration, as anaerobic metabolism becomes more prevalent. The increase in lactate production mirrors an increased activity in type II or fast twitch (FT) muscle fibres. According to Burke [29], the activation of these fibres is preferential once the AT has been reached. The increase in their activity explains the increase in muscular electrical activity, and supports the idea that the AT can be identified via the AT_EMG_.

### Possible control mechanisms

It is not clear which nervous centres are involved in producing and coordinating the responses to AT when this has been reached. However, these responses involve many organs, tissues and glands, and therefore many CNS structures are probably involved. The anatomo-functional relationships between the different structures of the encephalon preclude the recognition of any particular area of the brain as the centre governing the efferent signals involved in any response. However, in this response system, the brainstem and spinal cord are likely subordinate to the primitive structures of the pros encephalon. However, this paper uses the term central governor or central command (CC) to designate this functional entity [30].

The nervous centres and relationships between them that are involved in the processing of stress stimuli within the CNS are not well known. However, several hypotheses exist with respect to the role of the CC in fatigue [31], and different parts of the CNS (cortex, hippocampus, brainstem, encephalon, hypothalamus and amygdala) have been related directly or indirectly with the physiological response to stress [32]. The amygdala, which forms part of the limbic system, appears to have an important role in the latter. Further, its relationship with the hypothalamus and the brainstem suggest that this structure takes part in the activation and control of autonomic efferent activity. The nervous links between the limbic system and the hypothalamus, and then the brainstem nuclei, would appear to be responsible for sending efferent signals to responding tissues and organs. Since the peripheral sensory signals from mechanoreceptors, chemoreceptors and baroreceptors arrive instantly at the cardiac and respiratory control centres, the CC must deliver autonomic efferent responses that coincide in time.

Anatomically and electrophysiologically, the descending pathways that act directly or indirectly on the motor neurons of the anterior horn of the spinal cord include: 1) the corticospinal pathway, 2) the vestibulospinal (medial and lateral) pathways, 3) the reticulospinal (lateral and medial) pathways, 4) the rubrospinal pathway, 5) the interstitiospinal pathways, and 6) the tectoespinal pathway.

An increase in the activity of the descending motor pathways as a whole explains the increase in the activity of the FT fibres, and thus an increase in the lactate concentration. The efferent systems of the supraspinal centres communicate with motor neurones of different thresholds; they can therefore excite those with the highest thresholds (FT fibres) and inhibit those with the lowest (slow twitch [ST] fibres) [29]. It has also been suggested that populations of motor neurons may be activated differently, following different orders of recruitment [33]. This would allow the activation of an entire motor neurone population and avoid their saturation at a low level of activation, responding to spinal and supraspinal influences.

The electrophysiological characteristics of motor neurones, and the effect of the descending pathways on the pool of spinal motor neurons, explains the increase the activity of those innervating the FT once the AT has been reached [29].

### Physiological significance of the integrated physiological response to having reached the anaerobic threshold

The directing action of the CC allows for the coordination of the autonomous and motor systems, suggesting that the AT can be identified in the many - and apparently unrelated - ways described. The change in response to the AT having been reached probably allows the CNS to interpret that the organism is reaching its limits. At the same time, the different signals sent by the CNS may therefore also serve as a feedback system, as suggested by several authors [34]. Thus, increases in lactate, catecholamines and ventilation, etc. provide the higher centres related with consciousness with information that the organism is close to the VO_2_ max.

Of all the variables that are measurable during intense exercise, the HR has been the most studied over the last 20 years [35-37]. Although still a matter of debate, it is thought that the HR obeys a chaotic behavioural model [38]. Such behaviour may be accentuated once the AT has been reached [39,40]. The physiological response of an organism beyond the AT may also be chaotic. Such non-linear, chaotic behaviour explains why the response is limited to trying to maintain the balance between energy availability and use.

## Conclusion

In summary, the changes in the composition of the saliva, electromyographic activity, plasma catecholamines, and the other variables mentioned in this work, act as parts of a complex system that allows the CC to coordinate an effective response. An increase in load after reaching the AT means the organism must start to face homeostatic difficulties; the CC therefore sends information to all kinds of other control centres, many of which may not be directly linked to exercise. Thus, beyond the AT, the disorder increases for some variables (e.g., ventilation) or leads to a change in the slope away from linear (e.g., as seen for the HR). This change in response could be indicative that the organism would face failure if the load continued to increase. To avoid this, the CC manages the efferent signals that show the organism that it is running out of homeostatic potential. Unfortunately, we are a long way from knowing how the CC detects variations in these variables and coordinates its response.

## Competing interests

The authors declare that they have no competing interests.

## Authors’ contributions

ABP, JJR and FJC conception and design of this review; ABP and FJC drafted manuscript; JJR prepared figures; ABP, JJR and NM edited and revised manuscript; All authors read and approved the final manuscript.

## Pre-publication history

The pre-publication history for this paper can be accessed here:

http://www.biomedcentral.com/2052-1847/6/17/prepub
